# Carbolic Acid in Surgery

**Published:** 1893-05

**Authors:** 


### Carbolic Acid in Surgery.

There is considerable excitement in surgical circles just now owing to the recantation of Sir Joseph Lister in regard to the use of carbolic acid and mercuric salts in surgery. He has now abandoned the latter entirely, and has gone back to the former. The fact was mentioned in *The Chemist and Druggist* a few months ago (November 5, 1892), but the medical papers have just got hold of it through an address delivered by Sir Joseph at King's College the other week. It seems that the reason why corrosive sublimate began to displace carbolic acid was because Koch made some observations which showed that the former was more powerful than the latter; but it turns out that Koch exaggerated the power of the sublimate, and in such solutions as can be used in surgery it is much inferior to the acid in bactericidal power. Professor Crookshank has made experiments with the two antiseptics upon the tubercle bacillus, which further demonstrate the superiority of carbolic acid. So much for what may be called the theory. In practice Sir Joseph Lister uses a 1-in-20 solution of carbolic acid, and he states that while "it is more trustworthy as a germicide for surgical purposes than corrosive sublimate, it is in other respects also greatly to be preferred. Carbolic acid has a powerful affinity for the epidermis, penetrating deeply into its substance, and it mingles with fatty materials in any proportion. Corrosive sublimate solution, on the other hand, cannot penetrate in the slightest degree into anything that is greasy; and therefore, as the skin is greasy, those who use corrosive sublimate require elaborate precautions in the way of cleansing the skin—treating it with oil of turpentine or ether, not to mention soap and water, to remove the grease which they feel it essential to get rid of for the efficient action of the corrosive sublimate. All this is unnecessary care if you use carbolic lotion." It is satisfactory to note that the founder of Listerism has gone back to his old faith. It deserves to be mentioned that for several years the quantity of corrosive sublimate used in the principle hospitals of London has been on the decrease, and carbolic acid has been taking its place. The reason for this has not been exactly that put forward by Sir Joseph Lister, but the fact that mercurial poisoning was becoming very frequent amongst surgical patients. This was due, of course, to the corrosive sublimate being pushed too far, as, indeed, antisepism is apt to be. There is a growing belief that asepism rather than antisepism, will hold the surgical field in the future.—*The Chemist and Druggist.*

